# Randomised controlled community trial assessing efficacy of the AWACAN-ED public toolkit to improve cancer symptom awareness and intention to seek help in South Africa and Zimbabwe: study protocol

**DOI:** 10.1136/bmjopen-2025-106400

**Published:** 2026-01-14

**Authors:** Suzanne Scott, Jone G Lurgain, Sarah Day, Bothwell T Guzha, Ekaterina Pazukhina, Kirsten Deanne Arendse, Sudarshan Govender, Mike Chirenje, Valerie Anne Sills, Jane Harries, Rosemary Jacobs, Jennifer Moodley, Fiona M Walter

**Affiliations:** 1 Wolfson Institute of Population Health, Queen Mary University of London, London, UK; 2 School of Public Health, Faculty of Health Sciences, University of Cape Town, Cape Town, Western Cape, South Africa; 3 Faculty of Medicine & Health Sciences, University of Zimbabwe, Harare, Zimbabwe; 4 School of Public Health, Faculty of Health Sciences, University of Cape Town, Rondebosch, South Africa; 5 Department of Obstetrics, Gynaecology & Reproductive Sciences, University of California San Francisco School of Medicine, San Francisco, California, USA; 6 Cancer Association of South Africa, Johannesburg, South Africa; 7 UMTHA Strategy Planning and Development, Cape Town, South Africa; 8 Cancer Research Initiative, Faculty of Health Sciences, University of Cape Town, Rondebosch, South Africa; 9 The Primary Care Unit, Department of Public Health and Primary Care, University of Cambridge, Cambridge, UK

**Keywords:** Early Detection of Cancer, Awareness, Health Education, Randomized Controlled Trial, Primary Health Care, Africa South of the Sahara

## Abstract

**Introduction:**

Despite the benefits of early diagnosis, most cancers in sub-Saharan African (SSA) countries are diagnosed at an advanced stage due to late presentation of symptoms, inadequate referral systems and poor diagnostic capacity. Health communication interventions have been used extensively in high-income countries to increase people’s awareness of cancer symptoms and encourage timely help-seeking. However, in SSA, there is still limited evidence on the effectiveness of these interventions and existing evaluations are mainly focused on communicable diseases rather than cancer.

**Methods and analysis:**

A randomised, multisite, controlled community trial will evaluate a culturally tailored health infographic toolkit delivered in rural and urban settings in the Western Cape Province in South Africa and Harare and surrounding provinces in Zimbabwe. Participants will be randomised to receive one of three African aWAreness of CANcer and Early Diagnosis (AWACAN-ED) cancer awareness tools, coproduced with local communities, comprising health communication infographics with descriptions of breast, cervical and colorectal cancer symptoms plus messages to encourage consultation with primary care providers if symptoms occur, all presented in English and four local languages. We will recruit 144 participants in each of the three intervention groups (N=432). The primary outcome will be recall of symptoms and the secondary outcomes will be (1) intention to seek help, (2) emotional impact and (3) acceptability of the toolkit. Outcomes will be measured preintervention and at two points postintervention: after 15 min and 1 month.

**Ethics and dissemination:**

Ethical approval was obtained in both participating countries, South Africa (148/2025) and Zimbabwe (363/2021). All participants will be required to provide written informed consent prior to participation. Findings will be disseminated through peer-reviewed publications, conference presentations and the AWACAN-ED programme website.

**Trial registration number:**

PACTR202505475803308.

**STRENGTHS AND LIMITATIONS OF THIS STUDY**

- The study will test the psychological impact of culturally sensitive health messaging and infographics in English and four local languages in the African aWAreness of CANcer and Early Diagnosis (AWACAN-ED) public toolkit to investigate emotional response to engagement with the toolkit.
- Engagement of community members of different ages in the codesign and development of the intervention to enhance cultural appropriateness.
- Although each South African and Zimbabwean province was selected to be representative of rural and urban communities, they may have limited generalisability across the two countries and across sub-Saharan Africa.
- The trial will only measure the short-term impact of the AWACAN-ED public toolkit.

## Introduction

 Stage at diagnosis is a major determinant of cancer outcomes. In sub-Saharan African (SSA) countries, studies estimate that patients diagnosed with breast, cervical or colorectal cancer at an early stage (I/II) had 50%–62.5% 5-year survival probability compared with a 5-year survival of 20.5%–35.8% for those diagnosed at an advanced stage (III/IV).^1^,^3^ Despite early diagnosis leading to treatment with curative intent, most cancers in SSA are diagnosed at advanced stages. Studies estimate that in SSA, 64%, 66% and 70% of breast,^1^ cervical^2^ and colorectal^4^ cancers are diagnosed at an advanced stage, respectively. The main determinants of this advanced cancer diagnosis in SSA are late presentation of symptoms, inadequate referral systems and poor diagnostic capacity.^5^

The WHO recommends early diagnosis strategies for breast, cervical and colorectal cancers as a priority control measure in low-income and middle-income countries (LMICs), where cancer screening programmes are either not effectively implemented or non-existent.^6^ One strategy is to increase people’s awareness of symptoms and encourage timely presentation to healthcare professionals when symptoms occur.^7 8^

Health communication strategies aim to raise awareness of risk factors and symptoms, reinforce positive actions, influence social norms and change individual or group attitudes and behaviours.^9^ They can use different delivery formats, from conventional printed materials (eg, posters, leaflets, brochures) and broadcasting (eg, radio, television (TV), film) to digital messaging and social media (eg, X, Facebook, TikTok, WhatsApp). They can also use different design approaches from educational programmes and community outreach to printed or digital infographics. Successful health communication interventions will depend on how well contextual elements, such as linguistic and cultural differences, as well as health belief systems and values of the target population are incorporated in the design and implementation of the communication strategies.^10 11^ Thus, the development of health communication interventions must include extensive formative research, needs assessments and message pretesting.^9^

Although most evidence on health communication interventions aiming at increasing cancer awareness and encouraging help-seeking has been generated in high-income countries,^12 13^ there are examples of culturally tailored interventions conducted in LMICs with positive outcomes. For example, a successful health communication intervention to raise breast cancer awareness in India adopted a multilingual approach and segmented the target population into key homogenous groups based on religion and education levels.^14^ Whereas women with higher education levels were presented with a combination of facts and statistics related to breast cancer, women with lower education levels were given an educational programme using posters, presentations and leaflets adapted to their language and beliefs.^14^ Similarly, in Malaysia, another effective public awareness intervention for breast and colorectal cancer symptoms was culturally adapted for the three main ethnicities of the country. The intervention, a mass media campaign based on the UK’s ‘Be Cancer Aware’ programme and involving TV and radio advertisements and print materials (eg, billboards, posters), was adapted to the populations’ main languages (Malay, Chinese, Indian) and considered religious and other cultural values.^15^ In Africa, a systematic review, including evaluation studies in Nigeria, Tanzania, South Africa, Kenya, Malawi and Zambia, and another study conducted in Cameroon also demonstrated a positive impact of health education (eg, didactic lectures) and health communication (eg, information leaflets, videos) interventions on cervical cancer awareness.^16 17^ However, evidence on the effectiveness of these interventions in Southern Africa is still limited and the existing studies are mainly focused on addressing communicable diseases (COVID-19, HIV, Ebola), rather than cancer.^10^

This evaluation fills an essential gap by assessing the impact of research-informed and culturally tailored health communication interventions to improve awareness about cancer symptoms and to encourage timely help-seeking. The study is part of the NIHR-funded African aWAreness of CANcer and Early Diagnosis (AWACAN-ED) research programme, which aims to develop and evaluate tools for timely symptomatic diagnosis of cancer (https://awacan.online/). The overall aim of this study is to evaluate the efficacy of the AWACAN-ED public toolkit, which consists of a portfolio of locally tailored, context-sensitive and multilingual (English, Afrikaans, isiXhosa, Ndebele and Shona) infographics with health messaging delivered in both a rural and an urban setting in large regions in South Africa (Western Cape Province) and Zimbabwe (Harare and surrounding provinces). The primary objective is to test the impact of the exposure to the AWACAN-ED public toolkit on the recall of symptoms of breast, cervical and colorectal cancer. The secondary objectives are to examine the impact of the AWACAN-ED public toolkit on (1) the intention to seek help for symptoms of possible breast, cervical and colorectal cancer, (2) the emotional impact and (3) the acceptability of the toolkit.

## Methods and analysis

### Study design

The AWACAN-ED public toolkit study is a three-arm parallel-group randomised controlled multisite, community superiority trial. The study protocol reports in adherence with the Standard Protocol Items: Recommendations for Interventional Trials guideline.^18^,^20^ The main hypotheses of this randomised control trial (RCT) are that the exposure to the AWACAN-ED public tool will improve the recall of symptoms of breast, cervical and colorectal cancer and enhance the intention to seek timely help for symptoms of possible cancer, without increasing anxiety.

### Eligibility criteria and recruitment process

Participants will be eligible for the trial if they are at least 18 years old and reside in the selected study areas. Participants will be excluded if they fulfil any of the following exclusion criteria:

- Previous self-reported diagnosis of breast, cervical or colorectal cancer.
- Unable to communicate in English, Afrikaans, isiXhosa, Ndebele or Shona.
- Unable to provide informed consent.

The sample will be recruited in community-based sites, such as community centres, churches and at local events, with the assistance of members of the community advisory boards in the Western Cape (South Africa) and Harare and surrounding provinces (Zimbabwe). Previous AWACAN-ED health facility assessments informed the selection of these areas, which represent rural and urban communities and are comparable in size and population. Experienced field workers will approach potential participants, check eligibility, invite them to participate in the study and gain written informed consent. Both women and men will be included, but we will recruit more women than men (ratio 2:1). The inclusion of men in the evaluation of the cervical and breast cancer tools responds to the important role that male partners play in women’s help-seeking behaviour for cancer reported in the literature^21^ and was emphasised during our community workshops. Recruitment will last up to 6 months.

### Randomisation, intervention allocation and blinding

**Figure 1:**
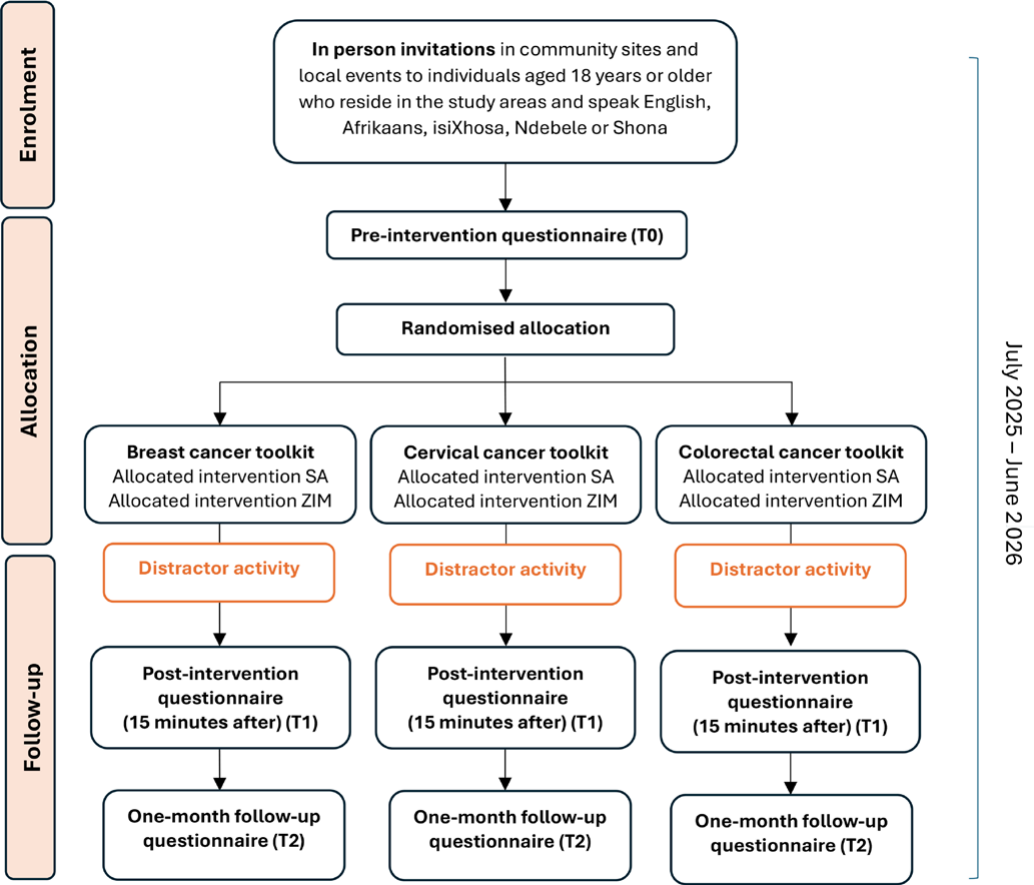
Participant flow chart based on the Consolidated Standards of Reporting Trials guidelines.

After consent is obtained, participants will be asked to complete a preintervention survey in their chosen language (English, Afrikaans, isiXhosa, Ndebele and Shona), including sociodemographics and any previous experience of breast, cervical and colorectal cancer among family and friends. Participants will then be randomly assigned to the exposure to one of the three (allocation ratio 1:1:1) cancer infographic tools (either breast, cervical or colorectal cancer). There will not be a ‘no intervention’ control group: each participant will only be exposed to one cancer tool and will act as the control group for the two other cancer tools.^22^ We have not included a ‘no intervention’ control group due to ethical concerns of withholding information about cancer symptoms to communities who are known to lack access to information and have low cancer awareness, but also due to the likelihood of participant demoralisation and attrition for taking part in a study but receiving no intervention. However, it is acknowledged that this prevents insight into the impact of multiple completions of the same survey. Simple randomisation techniques will be used in each of the four study settings using an online randomisation service (eg, www.sealedenvelope.com) (see study flow chart in figure 1). Due to the overt nature of the intervention, blinding participants and fieldworkers will not be possible, but data analysts will be blind to group and the allocation sequence will be concealed from the person allocating participants to the interventions using sealed opaque envelopes.

### Intervention

#### Patient and public involvement

**Figure 2:**
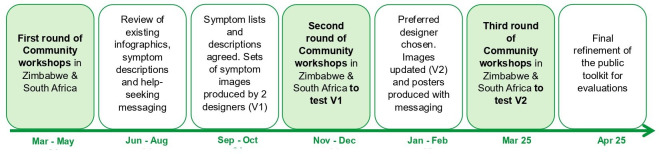
Codesign process of the AWACAN-ED public toolkit with communities in South Africa and Zimbabwe. AWACAN-ED, African aWAreness of CANcer and Early Diagnosis.

The AWACAN-ED programme includes two patient and public contributors (JH and RJ) who have been involved throughout its development and delivery, adding the perspective from those with lived experience of cancer. In addition, around 45 members of the communities (60% from the selected urban areas and 40% from the selected rural areas) in each of the two countries were involved in the intervention development. Following the Double Diamond design approach,^23^ representatives from the communities, such as religious leaders, teachers, local governmental officers, civil organisations and primary healthcare workers were invited to participate in three rounds of workshops with the research team to initially provide guidance on the focus of the intervention (Discovery and Define phases) and then provide feedback on the AWACAN-ED public toolkit design (Develop and Delivery phases), as well as on the dissemination plan. During the workshops, community participants discussed the images and wording of symptoms, including whether they were easy to understand and culturally appropriate, reactions to help-seeking messaging and reviews of translations of the tools into local languages (see the intervention development process in figure 2).

#### The AWACAN-ED public toolkit

The toolkit consists of health communication infographics with descriptions of possible symptoms of breast, cervical or colorectal cancer as well as messages to encourage consultation with primary care providers when symptoms occur. Each set of health infographics is available in English, Afrikaans, isiXhosa, Ndebele and Shona (five languages for three sets of cancer symptoms, producing a portfolio of 15 tools, available at http://www.awacan.online). Symptoms depicted in the AWACAN-ED public toolkit were adapted from WHO guidelines^24^,^26^ and other existing cancer awareness campaign materials. Commissioned graphic designers, facilitated by members of relevant cancer organisations, including the Cancer Association of South Africa (CANSA), were involved in developing prototype infographics for feedback from community participants.

#### Delivery of intervention

The intervention will be delivered face-to-face and one person at a time by experienced fieldworkers. Participants will be asked to complete a pre-intervention questionnaire (T0) and then to interact with and review the content of a printed version of their assigned cancer infographic for about 10 min. They will receive the tool in both English and their chosen language. After exposure to the tool, participants will be invited to take a break and to watch a video for around 15 min. This short activity, not related to health, will serve as a distractor item before participants are asked to complete the post-intervention questionnaire (T1).

### Outcome measures

The preintervention (T0) assessment will include sociodemographic questions (age, gender, relationship status, education and language most spoken at home), and any previous knowledge and experience of breast, cervical or colorectal cancer among family and friends.

To evaluate the efficacy of the AWACAN-ED public toolkit, we will measure two outcomes before (T0) and at two points after the intervention (T1: 15 min after the intervention; T2: 1 month after the intervention): recall of symptoms (primary outcome) and intention to seek help (secondary outcome). See study instrument in online supplemental material 1.

To assess recall of symptoms, an open-ended question will be used: *“Please would you name as many symptoms or signs of [breast, cervical, or colorectal] cancer as you can think of?”*.^27^ Responses will be recorded verbatim and will be coded by two researchers in each country against an agreed list of symptoms from the AWACAN survey for breast (15 symptoms) and cervical (11 symptoms) cancer awareness^27^ and from the Bowel Cancer Awareness Measure (9 symptoms).^28^ Participants will score ‘1’ if they mention a symptom that corresponds to the list of symptoms, and ‘0’ if they do not mention it and summed to produce total score of the number of recalled symptoms for each cancer type.

Intention to seek help will be measured by asking participants to report how soon they would visit the pharmacy or clinic or health centre or hospital, if they noticed symptoms of possible breast, cervical and colorectal cancer^27^ with five response options (‘never’, ‘less than 1 week’, ‘between a week and 1 month’, ‘between a month and 3 months’ and ‘3 months or more’). Only women will be asked the items about seeking help for breast or cervical symptoms. For analysis, responses will be dichotomised to ‘less than 1 week’ and ‘more than 1 week’.

To assess the emotional impact of the AWACAN-ED public tools, we will measure state anxiety which is defined as a transitory emotional response to a specific situation. A measure of emotional response to cancer symptoms^29^ that has previously been used in the African context was adapted for this study. The scale contains four items on feeling anxious, distressed, concerned and afraid. The participants select from three response options (‘no, not at all’, ‘yes, a bit’, ‘yes, a lot’) which are assigned numerical values (1–3). These values are summed to calculate an anxiety score. Higher scores indicate higher levels of state anxiety. This state anxiety measure will be administered prior to the intervention (T0) and in the first postintervention questionnaire (T1).

As an indicator of acceptability, participants will be asked how engaging they found the AWACAN-ED tool across five aspects of engagement (appealing, unpleasant, interesting, hard to understand, informative) adapted from previous studies.^30 31^ Response options will include ‘agree’; ‘disagree’ and ‘don’t know’. Participants will have the opportunity to add further comments or suggestions regarding the AWACAN-ED tool. Responses will be recorded verbatim. These items will only be asked in the first postintervention questionnaire (T1).

### Sample size and data analysis plan

Participant characteristics and outcomes will be summarised using descriptive statistics, including mean and median, SD and IQR. To assess how engaging participants found the health communication tools, we will use descriptive statistics to report the proportion of participants who agreed with each aspect of engagement. For the open response question, two researchers will independently code the responses into thematic categories.

We have estimated the sample size based on the assumption that the baseline number of expected recalled symptoms before the intervention will be equivalent to the average number of breast cancer symptoms recalled in South Africa using the same measure.^32^ The expected effect of the intervention on symptom recall was assumed to match or exceed the increase observed in a comparable study of lung cancer symptom recall in the UK^33^ that had a more conservative impact than a recent quasi-experimental study in South Africa.^34^ Based on these assumptions, a sample size calculation for a paired t-test with a significance level (α) of 0.05 and a power of 80% resulted in an estimated requirement of 144 participants per intervention group (N=432). This will be distributed across the two settings in South Africa and two in Zimbabwe (see table 1). To allow subgroup analyses by gender, age band and language, we will recruit at least 20 participants to each category and recruit twice as many women (n=288) as men (n=144). We anticipate minimal missing data as the questionnaires will be administered by experienced fieldworkers and researchers. However, listwise deletion will be used if any data is missing at random. Statistical analyses will be conducted using R software (2021).^35^

**Table 1:** Planned minimum sample sizes required and distribution by intervention group

Research site	Intervention groups			Total
	Breast cancer tool	Cervical cancer tool	Colorectal cancer tool	
South Africa (Western Cape)				
Urban	36	36	36	108
Rural	36	36	36	108
Zimbabwe (Harare)				
Urban	36	36	36	108
Rural	36	36	36	108
Total	144	144	144	432

### Data collection and management

Prior to the start of the study, the questionnaire items will be piloted in English with at least two participants in each community site in both countries (n=8). We will then translate and pilot the questionnaire items into Afrikaans, isiXhosa, Ndebele and Shona using the following steps^36^:

1. Forward and backward translations^37^ undertaken by professional translators and bilingual fieldworkers per each language.
2. ‘Expert’ review: AWACAN-ED team members and/or other cancer researchers fluent in both English and the local language will evaluate the translations and reconcile discrepancies.
3. Interviews will be conducted with local language speaking community participants using the Afrikaans and isiXhosa version (SA, n=4), and the Ndebele and Shona version (ZIM, n=4) to clarify meaning and understanding of each item.

Data will be collected by experienced fieldworkers using a hand-held electronic tablet customised with the questionnaire and loaded with the RedCap software. Participants will be assigned a participant ID code so that their data is not linked to their identity. For sociodemographic characteristics, participants will have the option to not answer questions related to their relationship status and education level. These cases will be treated as missing data.

The study team will meet weekly and the independent study steering committee will meet quarterly to monitor data. Any protocol modifications will be reported to the trial registry. On completion of the study, team anonymous digital data will be available from the University of Cape Town following an embargo period of 12 months after project completion.

## Discussion

To our knowledge, this is the first RCT across South Africa and Zimbabwe to evaluate the efficacy and acceptability of a cancer awareness communication intervention using locally tailored health infographics which combine pictures and text to depict symptoms of breast, cervical and colorectal cancer. The study will provide findings around the potential effect of these locally tailored health infographics in improving symptom awareness. This could produce insights for policy makers to incorporate into cancer prevention and early detection programmes and healthcare providers who could use these health infographics to better engage with patients by providing culturally appropriate information.

The major strength of the study is the community codesign of the intervention ensuring a culturally sensitive toolkit in five languages for local use across two Southern African countries, which may contribute to the acceptability of the tools as well as facilitate participant recruitment. The toolkit has been carefully designed to minimise anxiety and the current study will test this to provide evidence that campaigns using these resources are unlikely to cause distress. Although the study will evaluate the efficacy of the health infographics on cancer symptom awareness in some of the most commonly spoken languages in the research settings, future adaptations will be required to develop and evaluate the AWACAN-ED public toolkit for use in other regions with different local languages and cultural requirements to ensure contextual suitability, and in wider settings to ensure generalisability. Similarly, the toolkit could be developed for symptoms of other common cancers such as prostate, lung and oesophagus. The longer-term impact (beyond 1 month) and measurement of the effect on actual help-seeking behaviour is not possible in the time frame of this study. There are elements of this study that could be investigated further in future research, including whether participants seek information about other cancers after being exposed to information about one cancer. Further research studies will also be required to evaluate the impact of the toolkit on healthcare use and investigate the necessary efforts and health systems adaptations (eg, improving human resources and coordination between primary care and specialist levels) needed to respond to the future burden of breast, cervical and colorectal cancer in Southern Africa.

## Ethics and dissemination

### Ethics

Ethical approval was first obtained on 14 April 2025, reference number 148/2025 in South Africa (UCT) and then by the Ethics Committee of the University of Harare (Zimbabwe). This study has been categorised as low risk. There are no expected adverse events related to the intervention or research procedures. All participants will be required to provide written informed consent prior to participation, and they will receive R200/US$10 after data collection completion as compensation for the time given to the study. The trial was registered in the Pan African Clinical Trials Registry (ref num. PACTR202505475803308) on 28 May 2025.

### Dissemination

The study findings will be disseminated through peer-reviewed publications, conference presentations, community workshops and reports published on the AWACAN-ED programme website. Findings of the study, including feedback and suggestions from the community workshops regarding dissemination plans, will also inform future national public awareness campaigns, as well as potential tailored community-based behavioural interventions to reduce appraisal and help-seeking intervals for patients with symptoms of possible breast, cervical and colorectal cancer in South Africa and Zimbabwe. All future dissemination activities will be implemented in collaboration with communities, relevant cancer prevention organisations (eg, CANSA) and other clinical and non-clinical stakeholders (eg, primary healthcare workers, policy makers).

## Trial status

The recruitment of participants started on 21 July 2025 with completion due by June 2026.

## Supplementary material

10.1136/bmjopen-2025-106400 online supplemental file 1

## References

[1] Joko-Fru WY, Miranda-Filho A, Soerjomataram I (2020). Breast cancer survival in sub-Saharan Africa by age, stage at diagnosis and human development index: A population-based registry study. Int J Cancer.

[2] Sengayi-Muchengeti M, Joko-Fru WY, Miranda-Filho A (2020). Cervical cancer survival in sub-Saharan Africa by age, stage at diagnosis and Human Development Index: A population-based registry study. Int J Cancer.

[3] Gullickson C, Goodman M, Joko-Fru YW (2021). Colorectal cancer survival in sub-Saharan Africa by age, stage at diagnosis and Human Development Index: A population-based registry study. Int J Cancer.

[4] Bouter C, Bebington B, Maphosa S (2020). It’s contrary - comorbidity does not affect survival of South Africans with colorectal cancer: An analysis from the Colorectal Cancer in South Africa cohort. S Afr Med J.

[5] Martins T, Merriel SWD, Hamilton W (2020). Routes to diagnosis of symptomatic cancer in sub-Saharan Africa: systematic review. BMJ Open.

[6] World Health Organization (2020). WHO report on cancer: setting priorities, investing wisely and providing care for all. https://www.who.int/publications/i/item/who-report-on-cancer-setting-priorities-investingwisely-and-providing-care-for-all.

[7] World Health Organization (2017). Guide to cancer early diagnosis. http://apps.who.int/iris.

[8] World Health Organization (2010). Cancer – screening and early detection. Fact-sheets. https://www.who.int/europe/newsroom/fact-sheets/item/cancer-screening-and-early-detection-of-cancer.

[9] Rimal RN, Lapinski MK (2009). Why health communication is important in public health. Bull World Health Organ.

[10] Olaoye A, Onyenankeya K (2023). A systematic review of health communication strategies in Sub-Saharan Africa-2015-2022. Health Promot Perspect.

[11] Kreps GJ (2003). The impact of communication on cancer risk, incidence, morbidity, mortality, and quality of life. Health Commun.

[12] Lai J, Mak V, Bright CJ (2012). Reviewing the impact of 11 national Be Clear on Cancer public awareness campaigns. Int J Cancer.

[13] Austoker J, Bankhead C, Forbes LJL (2009). Interventions to promote cancer awareness and early presentation: systematic review. Br J Cancer.

[14] Kreps GL, Sivaram R (2008). Strategic health communication across the continuum of breast cancer care in limited-resource countries. Cancer.

[15] Schliemann D, Paramasivam D, Dahlui M (2020). Change in public awareness of colorectal cancer symptoms following the Be Cancer Alert Campaign in the multi-ethnic population of Malaysia. BMC Cancer.

[16] Makadzange EE, Peeters A, Joore MA (2022). The effectiveness of health education interventions on cervical cancer prevention in Africa: A systematic review. Prev Med.

[17] Sossauer G, Zbinden M (2014). Correction: Impact of an Educational Intervention on Women’s Knowledge and Acceptability of Human Papillomavirus Self-Sampling: A Randomized Controlled Trial in Cameroon. PLoS ONE.

[18] Chan A-W, Tetzlaff JM, Gøtzsche PC (2013). SPIRIT 2013 explanation and elaboration: guidance for protocols of clinical trials. BMJ.

[19] Calvert M, Kyte D, Mercieca-Bebber R (2018). Guidelines for Inclusion of Patient-Reported Outcomes in Clinical Trial Protocols: The SPIRIT-PRO Extension. JAMA.

[20] Calvert M, King M, Mercieca-Bebber R (2021). SPIRIT-PRO Extension explanation and elaboration: guidelines for inclusion of patient-reported outcomes in protocols of clinical trials. *BMJ Open*.

[21] McCutchan G, Weiss B, Quinn-Scoggins H (2021). Psychosocial influences on help-seeking behaviour for cancer in low-income and lower middle-income countries: a mixed-methods systematic review. BMJ Glob Health.

[22] Tock WL, Maheu C, Johnson NA (2022). Considerations of Control Conditions Designs in Randomized Controlled Trials of Exercise Interventions for Cancer Survivors. *Can J Nurs Res*.

[23] Design Council (2019). Framework for innovation: Design Council’s evolved Double Diamond. www.designcouncil.org.uk/our-work/skills-learning/tools-frameworks/framework-for-innovation-design-councils-evolved-double-diamond.

[24] World Health Organization (2024). Breast cancer. Fact-sheets. https://www.who.int/news-room/fact-sheets/detail/breast-cancer.

[25] World Health Organization (2024). Cervical cancer. Fact-sheets. https://www.who.int/news-room/fact-sheets/detail/cervical-cancer.

[26] World Health Organization (2024). Colorectal cancer. Fact-sheets. https://www.who.int/news-room/fact-sheets/detail/colorectal-cancer.

[27] Moodley J, Scott SE, Mwaka AD (2019). Development and validation of the African Women Awareness of CANcer (AWACAN) tool for breast and cervical cancer. PLoS ONE.

[28] Bowel CAM (adapted from generic CAM). Developed by University College London and Cancer Research UK. Generic CAM was developed jointly by University College London, Cancer Research UK, Kings College London, and Oxford University, (2007-08).

[29] Meechan G, Collins J, Petrie KJ (2003). The relationship of symptoms and psychological factors to delay in seeking medical care for breast symptoms. Prev Med.

[30] Marlow LAV, Nemec M, Vlaev I (2022). Testing the content for a targeted age‐relevant intervention to promote cervical screening uptake in women aged 50–64 years. British J Health Psychol.

[31] Comello MLG, Qian X, Deal AM (2017). Acknowledgment Correction of: Impact of Game-Inspired Infographics on User Engagement and Information Processing in an eHealth Program. J Med Internet Res.

[32] Moodley J, Constant D, Mwaka AD (2020). Mapping awareness of breast and cervical cancer risk factors, symptoms and lay beliefs in Uganda and South Africa. PLoS ONE.

[33] McCutchan G, Smits S, Ironmonger L (2020). Evaluation of a national lung cancer symptom awareness campaign in Wales. Br J Cancer.

[34] Dlamini SB, Sartorius B, Ginindza TG (2023). Pre- and post-intervention survey on lung cancer awareness among adults in selected communities in KwaZulu-Natal, South Africa: A quasi-experimental study. J Public Health Afr.

[35] R Core Team (R) (2021). A language and environment for statistical computing. https://www.R-project.org.

[36] Hall DA, Zaragoza Domingo S, Hamdache LZ (2018). A good practice guide for translating and adapting hearing-related questionnaires for different languages and cultures. Int J Audiol.

[37] Maneesriwongul W, Dixon JK (2004). Instrument translation process: a methods review. J Adv Nurs.

