# Supplementary material for: Randomised controlled community trial assessing efficacy of the AWACAN-ED public toolkit to improve cancer symptom awareness and intention to seek help in South Africa and Zimbabwe: study protocol
Source: BMJ Open. 2026 Jan 14;16(1):e106400. doi: 10.1136/bmjopen-2025-106400 (PMC12815121; doi:10.1136/bmjopen-2025-106400)
Supplement: online supplemental file 1 [file bmjopen-16-1-s001.pdf]

**Supplementary material 1. Study instruments**

# AWACAN-ED public toolkit evaluation – Survey (English version)

**SITE CODE** ☐☐

**INTERVIEWER CODE** ☐☐

## INTRODUCE YOURSELF

Hello, my name is ................................................ (name of the interviewer) and I am part of a research team from the [University of Cape Town/University of Zimbabwe]. We are evaluating a health communication toolkit, which includes images and messages to raise awareness of breast, cervical and bowel cancer in the community. We are interested in inviting healthy people who are 18 years old or older, who live in this community and can communicate in one the following languages: [*Afrikaans, isiXhosa*] or [*Ndebele and Shona*] and English. I would like to invite you to take part in the study, but first I need to check some information about yourself:

## CHECK ELIGIBILITY OF POTENTIAL PARTICPANT (eligibility screener)

1. Are you 18 years of age or older?
   1 = yes
   2 = no -> Stop
2. Do you live in [study area]?
   1 = yes
   2 = no -> Stop
3. Have you been diagnosed with breast, cervical or colorectal cancer previously?
   1 = yes -> Stop
   2 = no -> Proceed

## READ INFORMATION SHEET AND RECEIVE INFORMED CONSENT (docs attached)

**AWACAN-ED PUBLIC TOOLKIT – PRE-INTERVENTION QUESTIONNAIRE (T0)**

**SITE CODE** ☐☐

**INTERVIEWER CODE** ☐☐

**PARTICIPANT ID CODE** ☐☐☐☐☐☐☐

**DATE OF INTERVIEW** ☐☐/☐☐/☐☐☐☐

**READ: "Thank you for agreeing to take part in the study. To start, I am going to ask you some questions about yourself".**

**SECTION 1: SOCIO-DEMOGRAPHIC QUESTIONS**

| No. | Questions | Response options |
|---|---|---|
| 101. | What is your gender? | Female |
| | | Male |
| | | Other (please specify)…………………… |
| | | I would prefer not to say |
| 102. | How old are you?<br><br>*Interviewer note: If the participant does not know their age, ask for their date of birth, calculate their age and write it down.*<br><br>*If the participant either does not know their age/date of birth or if refuses to provide their age try to get an age bracket e.g. 30-35 years*<br><br>*If the participant cannot provide an age or age bracket code as "99"* | ……………… years<br><br>………………. age bracket |
| 103. | What is your current relationship status?<br><br>**READ OUT ALL OPTIONS AND ENTER ONLY ONE RESPONSE** | Married |
| | | Living together with a partner |
| | | Single |
| | | Separated/Divorced |
| | | Widowed |
| | | Did not answer |
| 104. | What is your highest level of education?<br><br>*Interviewer note: use school grade and standard guide to assist you* | No schooling |
| | | Primary incomplete |
| | | Primary complete |
| | | Secondary incomplete |

| | | |
|---|---|---|
| | **READ OUT ALL OPTIONS AND ENTER ONLY ONE RESPONSE** | Secondary complete |
| | | More than secondary |
| | | Did not answer |
| 105. | What is the main language spoken in your home?<br><br>**READ OUT ALL OPTIONS AND ENTER ONLY ONE RESPONSE** | English |
| | | Afrikaans |
| | | isiXhosa |
| | | Ndebele |
| | | Shona |
| | | Other (please, specify)<br>................................ |

**READ: "The next questions are about how you feel right now. For each question, please, respond as 'Yes, very much', or 'Yes, a bit' or 'No, not at all'.**

**SECTION 2: STATE ANXIETY**

| No. | Questions | Response options |
|---|---|---|
| 201. | Do you feel **anxious** right now?<br><br>**READ OUT ALL OPTIONS AND ENTER ONLY ONE RESPONSE** | Yes, very much |
| | | Yes, a bit |
| | | No, not at all |
| | | |
| 202 | Do you feel **afraid** right now?<br><br>**READ OUT ALL OPTIONS AND ENTER ONLY ONE RESPONSE** | Yes, very much |
| | | Yes, a bit |
| | | No, not at all |
| | | |
| 203 | Do you feel **distressed** right now?<br><br>**READ OUT ALL OPTIONS AND ENTER ONLY ONE RESPONSE** | Yes, very much |
| | | Yes, a bit |
| | | No, not at all |
| | | |
| 204 | Do you feel **concerned** right now?<br><br>**READ OUT ALL OPTIONS AND ENTER ONLY ONE RESPONSE** | Yes, very much |
| | | Yes, a bit |
| | | No, not at all |

**READ: "The next questions are about cancer awareness"**

**SECTION 3: BREAST, CERVICAL AND COLORECTAL CANCER SYMPTOM AWARENESS**

| No. | Questions | Response options |
|---|---|---|
| 301a | Have you ever heard of breast cancer?<br>***IF "NO" SKIP TO question 302a** | Yes |
| | | No |
| 301b | Does anyone in your family or a close friend have breast cancer now or in the past? (This could be your parent/sibling/daughter/son/grandparents). | Yes |
| | | No* |
| 302a. | Have you ever heard of cervical cancer?<br>***IF "NO" SKIP TO question 303a** | Yes |
| | | No* |
| 302b | | Yes |

| | | |
|---|---|---|
| | Does anyone in your family or a close friend have cervical cancer now or in the past? ( This could be your parent/sibling/daughter/son/grandparents). | No* |
| 303a. | Have you ever heard of colorectal/bowel cancer?<br>***IF "NO" SKIP TO section 4** | Yes<br>No* |
| 303b. | Does anyone in your family or a close friend have colorectal/bowel cancer now or in the past? (This could be your parent/sibling/daughter/son/grandparents). | Yes<br>No |

**SECTION 4: RECALL OF SYMPTOMS**

*Interviewer note: The following are open questions seeking to find out how many symptoms or signs of breast, cervical and colorectal cancer people may know.*

| No. | Questions | Response options |
|---|---|---|
| 401 | Please name as many symptoms or signs of **breast cancer** as you can think of?<br><br>**TYPE IN ALL THE SYMPTOMS OR SIGNS THE PARTICIPANT GIVES IN THE BLANK SPACE PROVIDED. TYPE IT EXACTLY AS THEY SAY IT.** | |
| 402. | Please name as many symptoms or signs of **cervical cancer** as you can think of?<br><br>**TYPE IN ALL THE SYMPTOMS OR SIGNS THE PARTICIPANT GIVES IN THE BLANK SPACE PROVIDED. TYPE IT EXACTLY AS THEY SAY IT.** | |
| 403. | Please name as many symptoms or signs of **colorectal/bowel cancer** as you can think of?<br><br>**TYPE IN ALL THE SYMPTOMS OR SIGNS THE PARTICIPANT GIVES IN THE BLANK SPACE PROVIDED. TYPE IT EXACTLY AS THEY SAY IT.** | |

**READ: "The next questions are about seeking help."**

**SECTION 5: HELP-SEEKING BEHAVIOUR**

| No. | Questions | Response options |
|---|---|---|
| 501a. FOR WOMEN ONLY | If you noticed **a breast or armpit lump or hardening, with or without pain,** how soon would you visit your local clinic or community health worker or hospital?<br><br>**READ OUT ALL OPTIONS AND ENTER ONLY ONE RESPONSE** | Never<br>< 1 week<br>≥ 1 week < 1 month<br>≥ 1 month < 3 months<br>≥ 3 months |
| 501b. FOR WOMEN ONLY | If you noticed **abnormal or bloody fluid from your nipple**, how soon would you visit your local clinic or community health worker or hospital? | Never<br>< 1 week<br>≥ 1 week < 1 month<br>≥ 1 month < 3 months |

| | | |
|---|---|---|
| | **READ OUT ALL OPTIONS AND ENTER ONLY ONE RESPONSE** | ≥ 3 months |
| 501c.<br>FOR WOMEN ONLY | If you noticed **a change in size, shape or look of your breast**, how soon would you visit your local clinic or community health worker or hospital?<br><br>**READ OUT ALL OPTIONS AND ENTER ONLY ONE RESPONSE** | Never<br>< 1 week<br>≥ 1 week < 1 month<br>≥ 1 month < 3 months<br>≥ 3 months |
| 501d.<br>FOR WOMEN ONLY | If you noticed **a change in shape or look of your nipple, such as it is being pulled inwards**, how soon would you visit your local clinic or community health worker or hospital?<br><br>**READ OUT ALL OPTIONS AND ENTER ONLY ONE RESPONSE** | Never<br>< 1 week<br>≥ 1 week < 1 month<br>≥ 1 month < 3 months<br>≥ 3 months |
| 501e.<br>FOR WOMEN ONLY | If you noticed **a change in breast skin , such as dimpling (like orange peel), redness or darkening,** how soon would you visit your local clinic or community health worker or hospital?<br><br>**READ OUT ALL OPTIONS AND ENTER ONLY ONE RESPONSE** | Never<br>< 1 week<br>≥ 1 week < 1 month<br>≥ 1 month < 3 months<br>≥ 3 months |
| | | |
| 502a.<br>FOR WOMEN ONLY | If you noticed **increased or foul-smelling vaginal discharge,** how soon would you visit your local clinic or community health worker or hospital?<br><br>**READ OUT ALL OPTIONS AND ENTER ONLY ONE RESPONSE** | Never<br>< 1 week<br>≥ 1 week < 1 month<br>≥ 1 month < 3 months<br>≥ 3 months |
| 502b.<br>FOR WOMEN ONLY | If you had **bleeding between periods, after sex or after periods have stopped permanently,** how soon would you visit your local clinic or community health worker or hospital?<br><br>**READ OUT ALL OPTIONS AND ENTER ONLY ONE RESPONSE** | Never<br>< 1 week<br>≥ 1 week < 1 month<br>≥ 1 month < 3 months<br>≥ 3 months |
| 502c.<br>FOR WOMEN ONLY | If you had **persistent pain in the back, legs, or pelvis**, how soon would you visit your local clinic or community health worker or hospital?<br><br>**READ OUT ALL OPTIONS AND ENTER ONLY ONE RESPONSE** | Never<br>< 1 week<br>≥ 1 week < 1 month<br>≥ 1 month < 3 months<br>≥ 3 months |
| 502d.<br>FOR WOMEN ONLY | If you had **pain during sex or vaginal discomfort**, how soon would you visit your local clinic or community health worker or hospital?<br><br>**READ OUT ALL OPTIONS AND ENTER ONLY ONE RESPONSE** | Never<br>< 1 week<br>≥ 1 week < 1 month<br>≥ 1 month < 3 months<br>≥ 3 months |
| 502e. | If you had **a loss of appetite**, how soon would you visit your local clinic or community health worker or hospital? | Never<br>< 1 week<br>≥ 1 week < 1 month |

| | | |
|---|---|---|
| FOR WOMEN ONLY | **READ OUT ALL OPTIONS AND ENTER ONLY ONE RESPONSE** | ≥ 1 month < 3 months |
| | | ≥ 3 months |
| | | |
| 503a. FOR WOMEN & MEN | If you had **bleeding from your bottom or blood in your stools**, how soon would you visit your local clinic or community health worker or hospital?<br><br>**READ OUT ALL OPTIONS AND ENTER ONLY ONE RESPONSE** | Never |
| | | < 1 week |
| | | ≥ 1 week < 1 month |
| | | ≥ 1 month < 3 months |
| | | ≥ 3 months |
| 503b. FOR WOMEN & MEN | If you had **discomfort in your stomach, such as cramps, pain or bloating that won't go away**, how soon would you visit your local clinic or community health worker or hospital?<br><br>**READ OUT ALL OPTIONS AND ENTER ONLY ONE RESPONSE** | Never |
| | | < 1 week |
| | | ≥ 1 week < 1 month |
| | | ≥ 1 month < 3 months |
| | | ≥ 3 months |
| 503c. FOR WOMEN & MEN | If you noticed **a change in bowel habits for over 3 weeks, such as going more often, looser stools, constipation**, how soon would you visit your local clinic or community health worker or hospital?<br><br>**READ OUT ALL OPTIONS AND ENTER ONLY ONE RESPONSE** | Never |
| | | < 1 week |
| | | ≥ 1 week < 1 month |
| | | ≥ 1 month < 3 months |
| | | ≥ 3 months |
| 503d. FOR WOMEN & MEN | If you **felt constantly tired and lacking energy, even with enough rest,** how soon would you visit your local clinic or community health worker or hospital?<br><br>**READ OUT ALL OPTIONS AND ENTER ONLY ONE RESPONSE** | Never |
| | | < 1 week |
| | | ≥ 1 week < 1 month |
| | | ≥ 1 month < 3 months |
| | | ≥ 3 months |
| 503e. FOR WOMEN & MEN | If you had **unexplained weight loss that is sudden or losing weight without trying**, how soon would you visit your local clinic or community health worker or hospital?<br><br>**READ OUT ALL OPTIONS AND ENTER ONLY ONE RESPONSE** | Never |
| | | < 1 week |
| | | ≥ 1 week < 1 month |
| | | ≥ 1 month < 3 months |
| | | ≥ 3 months |

**AWACAN-ED PUBLIC TOOLKIT – POST-INTERVENTION QUESTIONNAIRE (T1)**

**SITE CODE** ☐☐

**INTERVIEWER CODE** ☐☐

**PARTICIPANT ID CODE** ☐☐☐☐☐☐☐

**DATE OF INTERVIEW** ☐☐/☐☐/☐☐☐☐

**ALLOCATED INTERVENTION:** BREAST / CERVICAL / BOWEL

**ALLOCATED INTERVENTION:** English / Afrikaans / isiXhosa / Ndebele / Shona

**SECTION 1: STATE ANXIETY**

**READ: "The next questions are about how you feel right now. For each question, please, respond as 'Yes, very much', or 'Yes, a bit' or 'No, not at all'.**

| No. | Questions | Response options |
|---|---|---|
| T1_101. | Do you feel **anxious** right now?<br><br>**READ OUT ALL OPTIONS AND ENTER ONLY ONE RESPONSE** | Yes, very much<br>Yes, a bit<br>No, not at all |
| | | |
| T1_102 | Do you feel **afraid** right now?<br><br>**READ OUT ALL OPTIONS AND ENTER ONLY ONE RESPONSE** | Yes, very much<br>Yes, a bit<br>No, not at all |
| | | |
| T1_103 | Do you feel **distressed** right now?<br><br>**READ OUT ALL OPTIONS AND ENTER ONLY ONE RESPONSE** | Yes, very much<br>Yes, a bit<br>No, not at all |
| | | |
| T1_104 | Do you feel **concerned** right now?<br><br>**READ OUT ALL OPTIONS AND ENTER ONLY ONE RESPONSE** | Yes, very much<br>Yes, a bit<br>No, not at all |

**SECTION 2: RECALL OF SYMPTOMS**

**READ: "The next questions are about cancer awareness"**

| No. | Questions | Response options |
|---|---|---|
| T1_201. | Please name as many symptoms or signs of **breast cancer** as you can think of?<br><br>**TYPE IN ALL THE SYMPTOMS OR SIGNS THE PARTICIPANT GIVES IN THE BLANK SPACE PROVIDED. TYPE IT EXACTLY AS THEY SAY IT.** | |
| T1_202. | Please name as many symptoms or signs of **cervical cancer** as you can think of?<br><br>**TYPE IN ALL THE SYMPTOMS OR SIGNS THE PARTICIPANT GIVES IN THE BLANK SPACE PROVIDED. TYPE IT EXACTLY AS THEY SAY IT.** | |
| T1_203. | Please name as many symptoms or signs of **colorectal/bowel cancer** as you can think of?<br><br>**TYPE IN ALL THE SYMPTOMS OR SIGNS THE PARTICIPANT GIVES IN THE BLANK SPACE PROVIDED. TYPE IT EXACTLY AS THEY SAY IT.** | |

**SECTION 3: HELP-SEEKING BEHAVIOUR**

**READ: "The next questions are about seeking help".**

| No. | Questions | Response options |
|---|---|---|
| T1_301a. FOR WOMEN ONLY | If you noticed **a breast or armpit lump or hardening, with or without pain,** how soon would you visit your local clinic or community health worker or hospital?<br><br>**READ OUT ALL OPTIONS AND ENTER ONLY ONE RESPONSE** | Never |
| | | < 1 week |
| | | ≥ 1 week < 1 month |
| | | ≥ 1 month < 3 months |
| | | ≥ 3 months |
| T1_301b. FOR WOMEN ONLY | If you noticed **abnormal or bloody fluid from your nipple**, how soon would you visit your local clinic or community health worker or hospital?<br><br>**READ OUT ALL OPTIONS AND ENTER ONLY ONE RESPONSE** | Never |
| | | < 1 week |
| | | ≥ 1 week < 1 month |
| | | ≥ 1 month < 3 months |
| | | ≥ 3 months |
| T1_301c. FOR WOMEN ONLY | If you noticed **a change in size, shape or look of your breast**, how soon would you visit your local clinic or community health worker or hospital?<br><br>**READ OUT ALL OPTIONS AND ENTER ONLY ONE RESPONSE** | Never |
| | | < 1 week |
| | | ≥ 1 week < 1 month |
| | | ≥ 1 month < 3 months |
| | | ≥ 3 months |
| T1_301d. | If you noticed **a change in shape or look of your nipple, such as it is being pulled inwards**, how | Never |
| | | < 1 week |

| | | |
|---|---|---|
| FOR WOMEN ONLY | soon would you visit your local clinic or community health worker or hospital?<br><br>**READ OUT ALL OPTIONS AND ENTER ONLY ONE RESPONSE** | ≥ 1 week < 1 month<br>≥ 1 month < 3 months<br>≥ 3 months |
| T1_301e. FOR WOMEN ONLY | If you noticed **a change in breast skin, such as dimpling (like orange peel), redness or darkening,** how soon would you visit your local clinic or community health worker or hospital?<br><br>**READ OUT ALL OPTIONS AND ENTER ONLY ONE RESPONSE** | Never<br>< 1 week<br>≥ 1 week < 1 month<br>≥ 1 month < 3 months<br>≥ 3 months |
| | | |
| T1_302a. FOR WOMEN ONLY | If you noticed **increased or foul-smelling vaginal discharge,** how soon would you visit your local clinic or community health worker or hospital?<br><br>**READ OUT ALL OPTIONS AND ENTER ONLY ONE RESPONSE** | Never<br>< 1 week<br>≥ 1 week < 1 month<br>≥ 1 month < 3 months<br>≥ 3 months |
| T1_302b. FOR WOMEN ONLY | If you had **bleeding between periods, after sex or after periods have stopped permanently,** how soon would you visit your local clinic or community health worker or hospital?<br><br>**READ OUT ALL OPTIONS AND ENTER ONLY ONE RESPONSE** | Never<br>< 1 week<br>≥ 1 week < 1 month<br>≥ 1 month < 3 months<br>≥ 3 months |
| T1_302c. FOR WOMEN ONLY | If you had **persistent pain in your back, legs, or pelvis**, how soon would you visit your local clinic or community health worker or hospital?<br><br>**READ OUT ALL OPTIONS AND ENTER ONLY ONE RESPONSE** | Never<br>< 1 week<br>≥ 1 week < 1 month<br>≥ 1 month < 3 months<br>≥ 3 months |
| T1_302d. FOR WOMEN ONLY | If you had **pain during sex or vaginal discomfort**, how soon would you visit your local clinic or community health worker or hospital?<br><br>**READ OUT ALL OPTIONS AND ENTER ONLY ONE RESPONSE** | Never<br>< 1 week<br>≥ 1 week < 1 month<br>≥ 1 month < 3 months<br>≥ 3 months |
| T1_302d. FOR WOMEN ONLY | If you had **a loss of appetite**, how soon would you visit your local clinic or community health worker or hospital?<br><br>**READ OUT ALL OPTIONS AND ENTER ONLY ONE RESPONSE** | Never<br>< 1 week<br>≥ 1 week < 1 month<br>≥ 1 month < 3 months<br>≥ 3 months |
| | | |
| T1_303a. FOR WOMEN & MEN | If you had **bleeding from your bottom or blood in your stools**, how soon would you visit your local clinic or community health worker or hospital?<br><br>**READ OUT ALL OPTIONS AND ENTER ONLY ONE RESPONSE** | Never<br>< 1 week<br>≥ 1 week < 1 month<br>≥ 1 month < 3 months<br>≥ 3 months |

| | | |
|---|---|---|
| T1_303b. FOR WOMEN & MEN | If you had **discomfort in your stomach, such as cramps, pain or bloating that won't go away**, how soon would you visit your local clinic or community health worker or hospital?<br><br>**READ OUT ALL OPTIONS AND ENTER ONLY ONE RESPONSE** | Never<br>< 1 week<br>≥ 1 week < 1 month<br>≥ 1 month < 3 months<br>≥ 3 months |
| T1_303c. FOR WOMEN & MEN | If you noticed **a change in bowel habits for over 3 weeks, such as going more often, looser stools, constipation**, how soon would you visit your local clinic or community health worker or hospital?<br><br>**READ OUT ALL OPTIONS AND ENTER ONLY ONE RESPONSE** | Never<br>< 1 week<br>≥ 1 week < 1 month<br>≥ 1 month < 3 months<br>≥ 3 months |
| T1_303d. FOR WOMEN & MEN | If you **felt constantly tired and lacking energy, even with enough rest,** how soon would you visit your local clinic or community health worker or hospital?<br><br>**READ OUT ALL OPTIONS AND ENTER ONLY ONE RESPONSE** | Never<br>< 1 week<br>≥ 1 week < 1 month<br>≥ 1 month < 3 months<br>≥ 3 months |
| T1_303e. FOR WOMEN & MEN | If you had **unexplained weight loss that is sudden or losing weight without trying**, how soon would you visit your local clinic or community health worker or hospital?<br><br>**READ OUT ALL OPTIONS AND ENTER ONLY ONE RESPONSE** | Never<br>< 1 week<br>≥ 1 week < 1 month<br>≥ 1 month < 3 months<br>≥ 3 months |

**SECTION 4: ENGAGEMENT WITH THE TOOL**

**READ: Now I am going to read some statements to describe the cancer awareness information that you have just looked at. Please, can you tell me if you agree or disagree with each statement.**

| No. | Questions | Response options |
|---|---|---|
| T1_401. | The information was appealing<br><br>**READ OUT ALL OPTIONS AND ENTER ONLY ONE RESPONSE** | Agree<br>Disagree<br>Don't know |
| | | |
| T1_402. | The information was unpleasant<br><br>**READ OUT ALL OPTIONS AND ENTER ONLY ONE RESPONSE** | Agree<br>Disagree<br>Don't know |
| | | |
| T1_403. | The information was interesting<br><br>**READ OUT ALL OPTIONS AND ENTER ONLY ONE RESPONSE** | Agree<br>Disagree<br>Don't know |

| | | |
|---|---|---|
| T1_404. | The information was hard to understand<br><br>**READ OUT ALL OPTIONS AND ENTER ONLY ONE RESPONSE** | Agree<br>Disagree<br>Don’t know |
| | | |
| T1_405. | The information was informative<br><br>**READ OUT ALL OPTIONS AND ENTER ONLY ONE RESPONSE** | Agree<br>Disagree<br>Don’t know |
| | | |
| T1_406. | Do you have any suggestions of how the information could be improved?<br>**TYPE ANSWERS EXACTLY AS THEY SAY IT.** | |
| T1_407. | Is there anything missing from the information?<br>**TYPE ANSWERS EXACTLY AS THEY SAY IT.** | |

**AWACAN-ED PUBLIC TOOLKIT – 1-MONTH FOLLOW-UP QUESTIONNAIRE (T2)**

**SITE CODE** □□

**INTERVIEWER CODE** □□

**PARTICIPANT ID CODE** □□□□□□□

**DATE OF INTERVIEW** □□/□□/□□□□

**SECTION 1: RECALL OF SYMPTOMS**

**READ: "The next questions are about cancer awareness"**

| No. | Questions | Response options |
|---|---|---|
| T2_101. | Please name as many symptoms or signs of **breast cancer** as you can think of?<br><br>**TYPE IN ALL THE SYMPTOMS OR SIGNS THE PARTICIPANT GIVES IN THE BLANK SPACE PROVIDED. TYPE IT EXACTLY AS THEY SAY IT.** | |
| T2_102. | Please name as many symptoms or signs of **cervical cancer** as you can think of?<br><br>**TYPE IN ALL THE SYMPTOMS OR SIGNS THE PARTICIPANT GIVES IN THE BLANK SPACE PROVIDED. TYPE IT EXACTLY AS THEY SAY IT.** | |
| T2_103. | Please name as many symptoms or signs of **colorectal/bowel cancer** as you can think of?<br><br>**TYPE IN ALL THE SYMPTOMS OR SIGNS THE PARTICIPANT GIVES IN THE BLANK SPACE PROVIDED. TYPE IT EXACTLY AS THEY SAY IT.** | |

**SECTION 2: HELP-SEEKING BEHAVIOUR**

**READ: "The next questions are about seeking help".**

| No. | Questions | Response options |
|---|---|---|
| T2_201a. FOR WOMEN ONLY | If you noticed **a breast or armpit lump or hardening, with or without pain,** how soon would you visit your local clinic or community health worker or hospital? | Never |
| | | < 1 week |
| | | ≥ 1 week < 1 month |
| | | ≥ 1 month < 3 months |
| | | ≥ 3 months |

| | | |
|---|---|---|
| | **READ OUT ALL OPTIONS AND ENTER ONLY ONE RESPONSE** | |
| T2_201b.<br>FOR WOMEN ONLY | If you noticed **abnormal or bloody fluid from your nipple**, how soon would you visit your local clinic or community health worker or hospital?<br><br>**READ OUT ALL OPTIONS AND ENTER ONLY ONE RESPONSE** | Never<br>< 1 week<br>≥ 1 week < 1 month<br>≥ 1 month < 3 months<br>≥ 3 months |
| T2_201c.<br>FOR WOMEN ONLY | If you noticed **a change in size, shape or look of your breast**, how soon would you visit your local clinic or community health worker or hospital?<br><br>**READ OUT ALL OPTIONS AND ENTER ONLY ONE RESPONSE** | Never<br>< 1 week<br>≥ 1 week < 1 month<br>≥ 1 month < 3 months<br>≥ 3 months |
| T2_201d.<br>FOR WOMEN ONLY | If you noticed **a change in shape or look of your nipple, such as it is being pulled inwards**, how soon would you visit your local clinic or community health worker or hospital?<br><br>**READ OUT ALL OPTIONS AND ENTER ONLY ONE RESPONSE** | Never<br>< 1 week<br>≥ 1 week < 1 month<br>≥ 1 month < 3 months<br>≥ 3 months |
| T2_201e.<br>FOR WOMEN ONLY | If you noticed **a change in breast skin , such as dimpling (like orange peel), redness or darkening,** how soon would you visit your local clinic or community health worker or hospital?<br><br>**READ OUT ALL OPTIONS AND ENTER ONLY ONE RESPONSE** | Never<br>< 1 week<br>≥ 1 week < 1 month<br>≥ 1 month < 3 months<br>≥ 3 months |
| | | |
| 502a.<br>FOR WOMEN ONLY | If you noticed **increased or foul-smelling vaginal discharge,** how soon would you visit your local clinic or community health worker or hospital?<br><br>**READ OUT ALL OPTIONS AND ENTER ONLY ONE RESPONSE** | Never<br>< 1 week<br>≥ 1 week < 1 month<br>≥ 1 month < 3 months<br>≥ 3 months |
| T2_202b.<br>FOR WOMEN ONLY | If you had **bleeding between periods, after sex or after periods have stopped permanently,** how soon would you visit your local clinic or community health worker or hospital?<br><br>**READ OUT ALL OPTIONS AND ENTER ONLY ONE RESPONSE** | Never<br>< 1 week<br>≥ 1 week < 1 month<br>≥ 1 month < 3 months<br>≥ 3 months |
| T2_202c.<br>FOR WOMEN ONLY | If you had **persistent pain in your back, legs, or pelvis**, how soon would you visit your local clinic or community health worker or hospital?<br><br>**READ OUT ALL OPTIONS AND ENTER ONLY ONE RESPONSE** | Never<br>< 1 week<br>≥ 1 week < 1 month<br>≥ 1 month < 3 months<br>≥ 3 months |
| T2_202d. | If you had **pain during sex or vaginal discomfort**, how soon would you visit your local clinic or community health worker or hospital? | Never<br>< 1 week<br>≥ 1 week < 1 month |

| | | |
|---|---|---|
| FOR WOMEN ONLY | **READ OUT ALL OPTIONS AND ENTER ONLY ONE RESPONSE** | ≥ 1 month < 3 months<br>≥ 3 months |
| T2_202d. FOR WOMEN ONLY | If you had **a loss of appetite**, how soon would you visit your local clinic or community health worker or hospital?<br><br>**READ OUT ALL OPTIONS AND ENTER ONLY ONE RESPONSE** | Never<br>< 1 week<br>≥ 1 week < 1 month<br>≥ 1 month < 3 months<br>≥ 3 months |
| | | |
| T2_203a. FOR WOMEN & MEN | If you had **bleeding from your bottom or blood in your stools**, how soon would you visit your local clinic or community health worker or hospital?<br><br>**READ OUT ALL OPTIONS AND ENTER ONLY ONE RESPONSE** | Never<br>< 1 week<br>≥ 1 week < 1 month<br>≥ 1 month < 3 months<br>≥ 3 months |
| T2_203b. FOR WOMEN & MEN | If you had **discomfort in your stomach, such as cramps, pain or bloating that won't go away**, how soon would you visit your local clinic or community health worker or hospital?<br><br>**READ OUT ALL OPTIONS AND ENTER ONLY ONE RESPONSE** | Never<br>< 1 week<br>≥ 1 week < 1 month<br>≥ 1 month < 3 months<br>≥ 3 months |
| T2_203c. FOR WOMEN & MEN | If you noticed **a change in bowel habits for over 3 weeks, such as going more often, looser stools, constipation**, how soon would you visit your local clinic or community health worker or hospital?<br><br>**READ OUT ALL OPTIONS AND ENTER ONLY ONE RESPONSE** | Never<br>< 1 week<br>≥ 1 week < 1 month<br>≥ 1 month < 3 months<br>≥ 3 months |
| T2_203d. FOR WOMEN & MEN | If you **felt constantly tired and lacking energy, even with enough rest,** how soon would you visit your local clinic or community health worker or hospital?<br><br>**READ OUT ALL OPTIONS AND ENTER ONLY ONE RESPONSE** | Never<br>< 1 week<br>≥ 1 week < 1 month<br>≥ 1 month < 3 months<br>≥ 3 months |
| T2_203e. FOR WOMEN & MEN | If you had **unexplained weight loss that is sudden or losing weight without trying**, how soon would you visit your local clinic or community health worker or hospital?<br>**READ OUT ALL OPTIONS AND ENTER ONLY ONE RESPONSE** | Never<br>< 1 week<br>≥ 1 week < 1 month<br>≥ 1 month < 3 months<br>≥ 3 months |
