# Supplementary material for: Randomised controlled community trial assessing efficacy of the AWACAN-ED public toolkit to improve cancer symptom awareness and intention to seek help in South Africa and Zimbabwe: study protocol
Source: BMJ Open. 2026 Jan 14;16(1):e106400. doi: 10.1136/bmjopen-2025-106400 (PMC12815121; doi:10.1136/bmjopen-2025-106400)
Supplement: Supplementary file 2 [file bmjopen-2025-106400.reviewer_comments.pdf]

PEER REVIEW HISTORY

BMJ Open publishes all reviews undertaken for accepted manuscripts. Reviewers are asked to complete a checklist review form and are provided with free text boxes to elaborate on their assessment. These free text comments are reproduced below.

ARTICLE DETAILS

Title (Provisional)

A study protocol for a randomised controlled community trial assessing efficacy of the AWACAN-ED public toolkit to improve cancer symptom awareness and intention to seek help in South Africa and Zimbabwe

Authors

Scott, Suzanne; Lurgain, Jone G.; Day, Sarah; Guzha, Bothwell T; Pazukhina, Ekaterina; Arendse, Kirsten Deanne; Govender, Sudarshan; Chirenje, Mike; Sills, Valerie Anne; Harries, Jane; Jacobs, Rosemary; Moodley, Jennifer; Walter, Fiona M

VERSION 1 - REVIEW

**Reviewer**	**1**
**Name**	**Singleton, Anna**
**Affiliation**	**The Daffodil Centre, Faculty of Medicine and Health**
**Date**	**13-Oct-2025**
**COI**	**None**

Authors describe the co-creation and evaluation of a public health toolkit of infographics to improve cancer symptom awareness and intention to seek medical advice if symptoms arise. The trial protocol is well written, adheres to the SPIRIT guidelines and provides important immediate (post-intervention) and longer-term (1-month post intervention) evidence for the use of the toolkit. The gap in research was well described. The co-creation and trial methodology are well thought out and supported by evidence. Below are queries for consideration:

1. The study design states that this is a randomised controlled trial, and effectiveness will be determined within groups. Can the authors provide further insights into why there is no control group? Although the authors describe that each intervention group will be a control for the other, the infographics toolkit is greater than usual care for all groups. If no control group is included, how can authors reduce risks of biases that arise from multiple completions of the same survey?

2. Authors allude to the idea that if a participant receives an infographic about one cancer, that it equates to no information about the other cancers. If it’s not already included, it may

be worth considering asking a question in the 1-month post intervention regarding if participants sought out information about the other cancers. This cross-education could be very interesting.

3. Minor comment: The authors' community-based co-creation process was comprehensive. The authors may want to consider describing their co-creation process within the context of a co-creation/co-design framework or forming their methodology into a framework that could support similar research by other groups.

VERSION 1 - AUTHOR RESPONSE

**Reviewer**	Comment	Response from authors
**(Reviewer 1)**	1. The study design states that this is a randomised controlled trial, and effectiveness will be determined within groups. Can the authors provide further insights into why there is no control group? Although the authors describe that each intervention group will be a control for the other, the infographics toolkit is greater than usual care for all groups. If no control group is included, how can authors reduce risks of biases that arise from multiple completions of the same survey?	There is a control group in this study, but there isn't a 'no-intervention' control group. As the reviewer states, each intervention group will be a control for the other. This is stated in the methods (see page 6) and we have expanded this section to provide reasons for this.<br><br>The reviewer is right to point out that there is a risk of bias that arises from multiple completions of the same survey. We have added this as a limitation (see page 6).
	2. Authors allude to the idea that if a participant receives an infographic about one cancer, that it equates to no information about the other cancers. If it's not already included, it may be worth considering asking a question in the 1-month post intervention regarding if participants sought out information about the other cancers. This cross-education could be very interesting.	We agree that this would be interesting to monitor. As we have started data collection, including follow-ups we are unable to add this question but have added it as a suggestion in the discussion (see page 12-13).
	3. Minor comment: The authors' community-based co-creation process was comprehensive. The authors may want to consider describing their co-creation process within the context of a co-creation/co-design framework or forming their methodology into a framework that could support similar research by other groups.	Thank you for this suggestion. We have added further detail relating to the co-design framework (see page 8).
